# Zinc in Cellular Regulation: The Nature and Significance of “Zinc Signals”

**DOI:** 10.3390/ijms18112285

**Published:** 2017-10-31

**Authors:** Wolfgang Maret

**Affiliations:** Metal Metabolism Group, Departments of Biochemistry and Nutritional Sciences, School of Life Course Sciences, Faculty of Life Sciences and Medicine, King’s College London, Franklin-Wilkins Bldg, 150 Stamford St., London SE1 9NH, UK; wolfgang.maret@kcl.ac.uk; Tel.: +44-(0)-20-7848-4264; Fax: +44-(0)-20-7848-4195

**Keywords:** zinc, homeostasis, signalling, regulation

## Abstract

In the last decade, we witnessed discoveries that established Zn^2+^ as a second major signalling metal ion in the transmission of information within cells and in communication between cells. Together with Ca^2+^ and Mg^2+^, Zn^2+^ covers biological regulation with redox-inert metal ions over many orders of magnitude in concentrations. The regulatory functions of zinc ions, together with their functions as a cofactor in about three thousand zinc metalloproteins, impact virtually all aspects of cell biology. This article attempts to define the regulatory functions of zinc ions, and focuses on the nature of zinc signals and zinc signalling in pathways where zinc ions are either extracellular stimuli or intracellular messengers. These pathways interact with Ca^2+^, redox, and phosphorylation signalling. The regulatory functions of zinc require a complex system of precise homeostatic control for transients, subcellular distribution and traffic, organellar homeostasis, and vesicular storage and exocytosis of zinc ions.

## 1. Zinc in Enzymatic Catalysis, Protein Structure, and Regulation of Proteins

**Figure 1:**
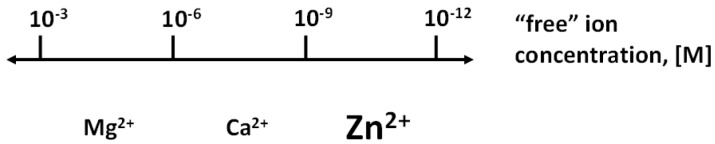
Biological regulation with the three redox-inert metals ions: Mg^2+^, Ca^2+^, and Zn^2+^. Zinc extends the range of regulation with metal ions. The regulatory function of each metal ion is in a specific range of concentrations, thus avoiding overlap with the signalling functions of the other metal ions. However, there are interactions among the metabolism of the metal ions.

It is ingrained in our understanding of the scientific literature that zinc has catalytic, structural, and regulatory functions in proteins. However, while the first two functions are well-established, validated examples of regulatory molecular functions are much more difficult to pinpoint. Catalytic and structural functions occur in an estimated 3000 human zinc metalloproteins, a number that translates into about every tenth protein being a zinc protein [1]. This myriad of functions demonstrates the major role of zinc in cell biology [2], which is now reinforced by the emerging roles of zinc ions in cellular regulation [3]. Many earlier postulates about regulatory functions of zinc in proteins are based on outdated premises, were not linked to specific molecular actions in physiological events, and involved zinc/protein interactions that occur with micromolar affinities. Compelling arguments put such interactions outside the physiological range of cellular zinc ion concentrations. They are based on experimental results and the requirement to control zinc in a range of concentrations that avoids interference with the biochemistry of the other essential metal ions. Measurements of zinc binding constants of zinc proteins, and total and available (“free”) zinc concentrations now provide a different view of the physiological significance of zinc/protein interactions. Affinities of structural and catalytic zinc in cellular proteins are in the picomolar to femtomolar range, and at present, evidence is lacking that these sites are regulated via zinc binding and release [4,5]. As a consequence of this high affinity, the “free” zinc ion concentration is in the picomolar range, as shown experimentally, despite the total cellular zinc concentration being in the range of hundreds of micromolar [6]. This large difference between total and “free” zinc is a distinctive chemical property of zinc in biology [7]. It rules out low affinity zinc binding sites as being physiologically significant for cellular regulation, and suggests that regulatory sites of zinc in cellular proteins must have binding constants commensurate with these chemical and biological constraints. In fact, a couple of examples of zinc inhibiting enzymes with picomolar affinity are now known [8,9,10]. Also, it was reported about 50 years ago that picomolar concentrations of zinc (II) ions inhibit phosphoglucomutase by replacing the magnesium ion required for activity [11]. The authors discussed the physiological significance of the finding, but their reports received very little, if any recognition in the field of metallobiochemistry [12]. In terms of its rather high affinity for binding sites, Zn^2+^ is different from Mg^2+^ and Ca^2+^, and makes an additional range available for biological regulation using all three redox-inert metal ions (Figure 1). What is needed for regulation is a change in the concentration of “free” zinc ions (zinc transients). This article discusses how such changes can be effected, and controlled, in a zinc-buffered biological environment. In the cell, zinc transients occur from a basal level of tens to hundreds of pM free zinc (about 0.2 mM total cellular zinc). They leave a gap of three orders of magnitude before calcium transients can set in, which are above a basal level of 100 nM free calcium (about 2 mM total cellular calcium). Like calcium, zinc needs a system to control these transients, and is cytotoxic if not properly controlled. Regarding magnesium, recently described circadian rhythms of total magnesium concentrations in cells provide strong evidence for regulatory functions [13].

## 2. Control of Cellular Zinc Homeostasis

**Figure 2:**
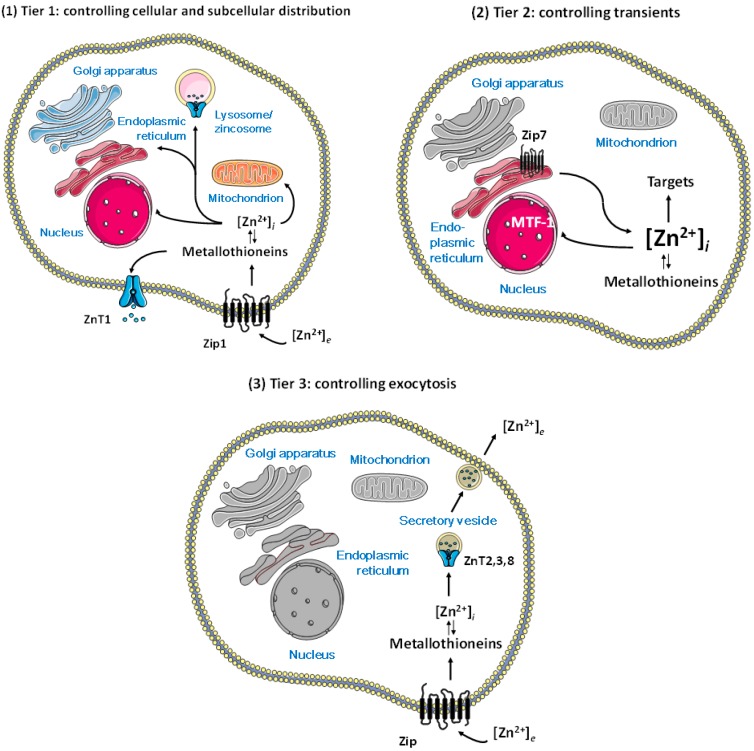
Control of cellular zinc homeostasis including regulation of zinc and functions of zinc in regulation. (**1**) Tier 1: cellular homeostasis through import and export through the plasma membrane and subcellular distribution; (**2**) Tier 2: vesicular storage and/or release of zinc associated with zinc importers and exporters on the vesicles; (**3**) Tier 3: loading of secretory vesicles with zinc associated only with zinc exporters on the vesicles. Regulatory functions of zinc require additional complexity in homeostatic control, namely, molecules that control the spatiotemporal fluctuations of zinc ions in a metal-buffered environment, above the fluxes in re-distributing zinc for supply as a cofactor in catalytic and structural sites of proteins. With this distinction, we assume that the functions of bona fide zinc proteins are not regulated by zinc coming on and off from their catalytic and structural sites. (Figures were composed from Servier Medical Art templates (http://smart.servier.com/)).

Knowledge about the proteins that control cellular zinc now provides a basis for understanding how zinc ions can regulate cellular processes. In humans, at least twenty-four membrane transporters (14 Zrt, Irt-like proteins (ZIP) zinc importers and 10 zinc transporters (ZnT) zinc exporters), about a dozen metallothioneins (MTs), and a zinc-sensing transcription factor, metal-response element (MRE)-binding transcription factor-1 (MTF-1), are involved in controlling cellular zinc [14]. A few of these transporters have a role—or an additional role—in Mn^2+^ and Fe^2+^ transport, while MT also has a function in copper metabolism [15]. The number of membrane transporters is much higher than what one would expect for simple control of homeostasis of a metabolite. Cellular iron, for example, is essentially controlled by one importer (DMT1) and one exporter protein (ferroportin). It turned out that many additional transporter proteins are necessary for the subcellular distribution of zinc, the control of organellar zinc homeostasis, and the generation and control of zinc transients. Most of the ZIP transporters have been found on the plasma membrane, but some have roles intracellularly. The large number of ZIP transporters on the plasma membrane is likely a reflection of the importance of securing the correct supply of zinc for the cell under various conditions. Remarkably, zinc ions accumulate in cellular vesicles for storage and/or release, and in secretory vesicles for exocytosis. The occurrence of zinc ions in cellular vesicles is a characteristic feature of cellular zinc biology, and resolves the long-standing issue why a storage protein akin to ferritin, which stores several thousand iron ions in its core, was never found for zinc. There is some ambiguity in the nature of some of these vesicles. Vesicular stores that accumulate zinc added to cultured cells have been called zincosomes [16]. Others are secretory vesicles from which zinc ions are exocytosed for various purposes. These vesicles differ from intracellular vesicular/organellar stores of zinc, as ZIP transporters do not seem to counteract the action of the ZnT transporters (ZnT2, 3, and 8) loading these vesicles with zinc ions. The “lethal milk” mouse has a truncated form of ZnT4, and presents with low zinc concentration in the milk and developmental defects of the mammary gland [17]. Zinc transporters ZnT4–7 are involved in loading zinc-requiring ectoenzymes with zinc at the *trans* Golgi network (TGN)/early secretory pathway [18]. Zinc is also translocated to lysosomes, mitochondria, and nuclei, but very little is known about the transporters involved. Zincosomes and related vesicles require additional components for control of zinc homeostasis in order to link cellular uptake and store loading. Thus, there is an at least three-tiered system for the homeostatic control of cellular zinc: import and export through the plasma membrane, including cytosolic binding proteins such as metallothioneins and sensors such as MTF-1 (tier 1); intracellular storage and release of zinc generating zinc transients (tier 2); and allocation of zinc for exocytosis in some cells, somehow linked to re-uptake (tier 3) (Figure 2). It appears that a hierarchy is associated with these three tiers, namely, that the capacity of loading vesicles is compromised, first, under zinc deficiency, as suggested by investigations with cultured cells [19].

**Figure 3:**
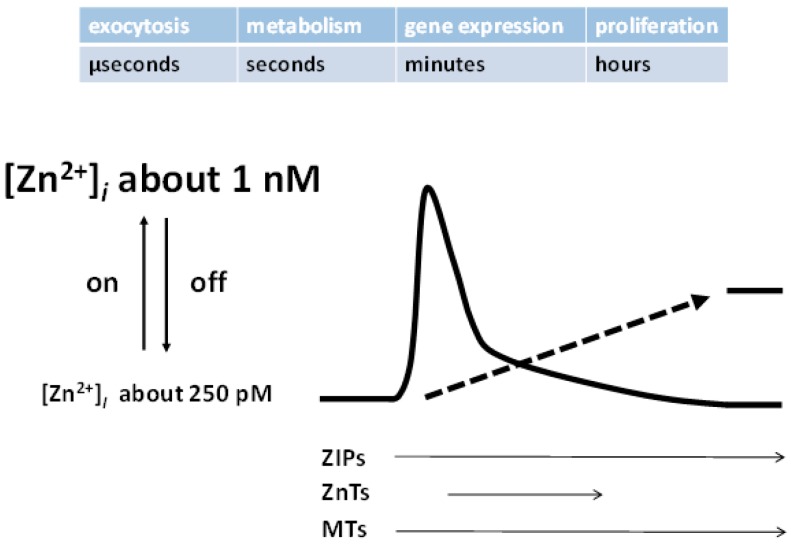
Cellular zinc transients and their control. Several pathways generate rapid zinc transients (zinc signals) with biological responses on various time scales. The amplitude of these zinc signals is about a few nanomolar globally, from picomolar basal “free” zinc ion concentrations, but may be higher in microdomains. Proteins involved in the control of zinc homeostasis serve as mufflers (ZIPs, ZnTs) and buffers (MTs) to restore the steady-state. A much slower process (arrow) leads to an overall change of the zinc-buffering characteristics of the cell, establishing different basal levels of “free” zinc ion concentrations.

There are at least three pathways for transient release of zinc ions within and from cells. The proteins participating in the control of homeostasis (ZIPs, ZnTs, metallothioneins, and MTF-1) restore the steady-state after the transients have occurred (Figure 3). A breakthrough that triggered the discovery of these pathways, and the functions associated with them, came with the synthesis and availability of fluorescent chelating agents for measuring zinc ions, similar to the ones employed in the field of calcium biology to measure calcium fluxes [20].

## 3. The Paradigms for Using Zinc Ions in Information Transfer/Regulation/Communication

A long train of discoveries followed the original observation of a histochemically stainable pool of zinc ions in the brain over half a century ago [21]. It culminated in the demonstrations that (i) the zinc ions localize to presynaptic vesicles (boutons) of specialized neurons; (ii) the zinc transporter ZnT3 loads the vesicles with zinc; and (iii) the zinc ions are exocytosed upon electrical stimulation of such neurons. Subsequently, three types of “zinc signals” in the brain have been defined: the synaptic signal, described as a type of chemical transmitter/neuromodulator; the transmembrane signal, where the zinc ions are translocated through the plasma membrane; and the intracellular zinc signal, with release of zinc ions from a source within cells [22]. All of these signals have now been shown to have physiological roles, not only in the brain, but importantly, in many other tissues, thus establishing at least three general pathways for the generation of zinc ion transients.

### 3.1. Release of Zinc Ions from Cells by Vesicular Exocytosis

Every cell has the capacity to export an excess of zinc. It appears that zinc is sequestered first in cellular vesicular stores, and that export from the cell occurs only after the capacity of the stores is exhausted. However, a number of cells, including neurons with glutamatergic/zincergic synaptic vesicles, have the additional capacity to secrete zinc ions by calcium-dependent exocytosis for a specific purpose [23,24]. In neurons, in addition to postsynaptic effects, zinc has a presynaptic effect, re-entering the neuron and affecting phosphorylation signalling via inhibition of protein tyrosine phosphatases (PTPs) [25,26]. In cells secreting zinc via exocytosis, there is an additional demand for zinc, as zinc brought into the secretory vesicles can reach relatively high concentrations (about mM) and needs to be replenished after secretion. High zinc concentrations have a function within the vesicles, e.g., a structural role together with calcium in the insulin hexamer in the dense granules of β-cells in the endocrine pancreas or inhibiting proteases in zymogen granules of acinar cells in the exocrine pancreas. Zinc ions are co-secreted from these vesicles, and paracrine, autocrine, and even endocrine roles for the zinc ions [Zn^2+^]_e_ have been suggested, e.g., in β-cells, in addition to their function in preventing amyloidosis of the secreted amylin and insulin [27]. For an endocrine effect, proof is lacking that the secreted cellular zinc increases the rather high total zinc concentration of blood sufficiently, in order for the liver to detect a zinc signal emanating from the pancreas [28]. It would seem that a specific complex of zinc needs to carry the information of the endocrine signal. Zinc-secreting cells also include prostate epithelial cells (zinc in prostate fluid), mammary gland epithelial cells (zinc in milk), intestinal Paneth cells, cells of the immune system (mast cells, granulocytes, neutrophils), and platelets. It is not clear whether zinc is the only cargo in any of these exocytotic vesicles. In fact, it is highly unlikely because secretory vesicles are biochemically quite complex, as shown for the dense granules of β-cells, which contain more than 300 different proteins [29]. It is also not clear how zinc is made available in the cytosol, where the concentration of free zinc is only picomolar, for loading the vesicles, and what the chemical forms of zinc are, in addition to protein-bound zinc in the vesicles.

Zinc in secretory vesicles → Ca^2+^-dependent exocytosis → increase of [Zn^2+^]_e_ → multiple targets

Another important biological event is the release of zinc ions from fertilized mouse oocytes [30]. These “zinc sparks” are preceded by calcium oscillations after fertilization/chemical-induced activation of the oocyte. The secreted zinc ions have a role in hardening the zona pellucida (glycoprotein matrix) to avoid polyspermy—yet another function of the secreted zinc on proteins.

### 3.2. Intracellular Release of Zinc Ions through Reactive Species Modifying Proteins

This pathway is based on the recognition that zinc coordination environments with thiolate (cysteine) sulphur, such as in MTs, are redox-active, despite zinc ions being redox-inert in biology. Oxidation of the sulphur ligand donor controls the dissociation of zinc ions [31]. It was the first pathway to show a way of releasing zinc intracellularly and increasing the free zinc ion concentrations, [Zn^2+^]_i_, overcoming the paradox of how zinc can be made available when it is bound with such high affinity to proteins. The list of oxidants is extensive, and includes reducible sulphur and selenium compounds [32,33]. In addition, reactive species such as nitric oxide, and electrophiles such as carbonyls react with the sulphur ligand donor [34]. The release of zinc has been shown in many cellular systems, e.g., nitric oxide signals target MTs and release zinc [35,36], and cell-permeable disulphides increase the available cellular zinc ion concentration [37]. The majority of investigations has addressed pathophysiological (oxidative stress releasing zinc), pharmacological (drugs releasing zinc), and toxicological (toxins releasing zinc) events. The released zinc ions first have a cytoprotective, and then a cytotoxic effect [38]. Many of the agents investigated react with MTs with concomitant release of zinc, which then activates MTF-1 to induce gene expression. In this pathway, MT is a signal transducer, generating zinc signals in response to redox signals. The physiological significance seems to be that signalling with many growth factors generates reactive species that can then generate cellular zinc transients [Zn^2+^]_i_ for the purpose of modulating signal transduction.

Reactive species/redox signalling → MTs → increase of [Zn^2+^]_i_ → multiple targets and MTF-1-dependent gene expression

### 3.3. Intracellular Release of Zinc Ions through Channels of Vesicular Stores

It was summarized, at the turn of the millennium, that zinc has functions at all levels of cellular signalling, and that growth factor signalling affects intracellular zinc re-distribution and functions [39]. The underlying idea, though, was that the extracellular zinc taken up by the cell is responsible for the observed effects.

A key discovery that expanded the possibilities of zinc as a signalling ion was that cross-linking of the high affinity immunoglobulin E receptor (Fcε receptor I) in mast cells causes release of zinc ions intracellularly from the perinuclear area, including the endoplasmic reticulum (ER). The response was dependent on calcium influx, caused mitogen-activated protein kinase (MAPK)/extracellular signal-regulated kinase (ERK) activation, and was called a “zinc wave”. While the targets of zinc were not identified, it demonstrated that zinc serves the role of a second messenger in modulating the signalling of plasma membrane receptors [40].

Another key observation was that zinc ions are released from an ER store through a specific pathway that includes the casein kinase 2-mediated phosphorylation and opening of the ZIP7 channel [41]. The released zinc affects phosphorylation signalling, and it appears to do so primarily by inhibiting PTPs [42].

Growth factor signals → casein kinase 2 (CK2) → ZIP7 phosphorylation → increase of [Zn^2+^]_i_ → inhibition of protein tyrosine phosphatases (PTPs) and enhanced phosphorylation signalling

## 4. Targets of Zinc Signals (Sensors, Effectors, and Stimuli)

Zinc binds to and stimulates a zinc-sensing receptor (ZnR) that triggers intracellular calcium release [43]. This receptor turned out to be the G-protein coupled receptor GPR39 [44,45,46], which activates inositol triphosphate (IP_3_) signalling and calcium release. What is not resolved, as yet, is where the zinc signal originates from, i.e., how a zinc signal can occur in a zinc-buffered extracellular milieu, whether it is part of the exocytotic pathway of zinc ions, and whether it originates from the same cell or another cell. GPR39 is an orphan receptor, and it is not clear whether or not zinc is the primary agonist or merely a modulator.

[Zn^2+^]_e_ → GPR39 → IP_3_ → increase of [Ca^2+^]_i_ → phosphorylation signalling (ERK/protein kinase B (AKT))

With the exception of ZnR/GPR39 and activation of MTF-1, the investigated effects downstream of the signalling zinc ions are, in most cases, not the zinc-dependent proteins themselves. Therefore, the direct molecular targets of the signalling zinc ions remain poorly defined. This is an important missing piece of information that is affecting the wider acceptance of Zn^2+^ as a second messenger, and so is the issue in which chemical form zinc ions transmit the signal, in particular, since a zinc-binding messenger protein, such as the calcium signal transducer calmodulin, has not been identified. In none of the cases of zinc signalling is the chemical identity of the zinc signal known. It is an important issue as the bioinorganic chemistry of zinc is quite different from that of calcium, and the term “free” does not apply in the same way. Any ligand of zinc could impact the specificity of the zinc signal, and thus, its information content [7]. Links between signals and effectors (targets) have been established, though. In neuronal zinc signalling, the NMDA (*N*-methyl-d-aspartate) receptor, a calcium channel, is a well characterized target. Zinc inhibits this receptor with nanomolar affinities at a structurally characterized site [47]. In the case of GPR39, calcium signalling is a downstream event of the zinc signal, too. Calcium signals can also be upstream of the zinc signal, e.g., when Ca^2+^/calmodulin activates nitric oxide synthase, and nitric oxide releases zinc from proteins. The intracellularly released “zinc wave” modulates phosphorylation signalling. Here, it seems that protein tyrosine phosphatases (PTPs) are targeted. Their inhibition enhances the phosphorylation signalling, i.e., the effect of kinases. PTPs are not described as metalloproteins, but zinc inhibits them with picomolar to nanomolar affinities with a specific mechanism that is independent of their redox modulation [48]. In the case of ZIP7 channel-released zinc, the phosphorylation signal is upstream and downstream of the zinc signal. There are other targets of zinc transients, and we are far from having a complete list of them [10]. The zinc signals downstream of redox signals seem to be a response to redox or carbonyl stress, and are linked to repair and defence pathways, and to initiating a response that restores the redox balance. PTPs are targets, but there is the potential for a much greater number of targets, depending on the intensity of the stress signal, and hence, the amplitudes of the zinc signal. Here, too, like in calcium and phosphorylation signalling, zinc transients/signals are upstream and downstream of redox effects, as zinc ions have pro-antioxidant and pro-oxidant effects, depending on their intracellular concentrations [49]. The observation of both upstream and downstream effects of zinc ion signals in signal transduction by calcium, redox, and phosphorylation signalling, suggests that the regulatory function of zinc signals is one of feedforward or feedback on these pathways and other pathways [50,51]. Zinc inhibition of phosphodiesterases controlling cAMP and cGMP signalling have been described, expanding this network of zinc regulation even further [39]. It appears that zinc, as the redox-inert metal ion with the highest affinity, is well suited for a regulatory position higher up in the hierarchy.

It remains crucial to quantitate the amplitudes and duration of zinc signals, and match them to the affinities of targets of zinc, in order to tease apart the biological significance of the signalling effects. Proteins targeted by transient zinc signals need to be differentiated from proteins affected by long-lasting changes in cellular zinc concentrations. In one case, the effect of the signal is readily reversible, while it will be long-lasting when the cell is brought into a different physiological state. Importantly, such a transition does not necessarily require a change in total zinc concentrations, but a change in the zinc buffering capacity, such as an increase or decrease in the concentrations of MTs. The very high affinity of some PTPs for zinc suggests that they are inhibited tonically, and then activated [9]. It is not known whether this occurs via a general mechanism, with generation of higher chelating capacity of the zinc-buffering species, or by specific molecules that bind to PTPs and remove the inhibitory zinc ion.

## 5. Genomic Effects and the Roles of Metallothioneins (MTs) and MTF-1 in Buffering and Muffling Zinc

**Figure 4:**
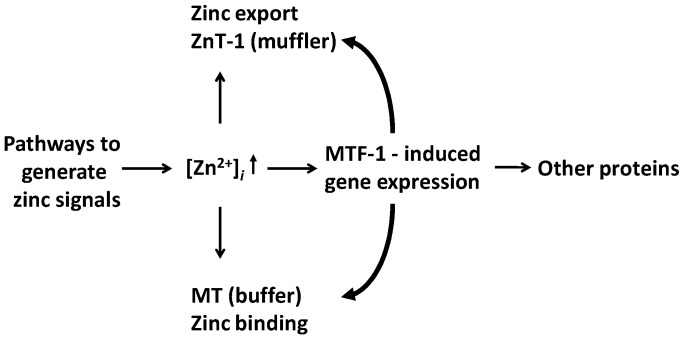
Genomic effects as a consequence of sensing zinc signals. MTF-1 induces the expression of the zinc exporter ZnT1 (muffler) and MT (buffer), which adjust the cellular zinc buffering capacity, and other proteins. In addition, constitutively expressed zinc transporters and MTs control the zinc signals.

Zinc signalling and zinc regulation are two different aspects. The discussed pathways of non-genomic zinc signalling eventually lead to genomic (transcriptional) responses via their effects on classical signal transduction pathways. These responses are part of zinc regulation. MTF-1 can sense zinc signals directly and induce gene transcription. More than 1000 genes have *cis*-acting metal response elements (MREs), but only 43 were identified as putative MTF-1 targets [52,53]. Among these targets, about 50% are transcription factors, and 19 are genes involved in development, demonstrating the role of zinc in developmental programmes as genetic ablation of MTF-1 is embryonically lethal in the mouse. Other target genes are γ-glutamate-cysteine ligase heavy chain needed for glutathione biosynthesis, and the zinc exporter ZnT1, and most, but not all MTs. In addition to the constitutively expressed ZnT1 and MT proteins buffering zinc signals/transients, the induced ZnT1 and MT proteins can adjust the zinc buffering capacity, and hence, the free zinc ion concentrations (Figure 4). Two effects are responsible for such adjustments. One is zinc buffering by MTs, and the other is a process called muffling, which refers to transporting metals into and out of the cytosol, and is a typical component of biological buffering mechanisms [54]. Knockdown of MTF-1 results in more genes becoming zinc-responsive and in unmasking repression of transcriptional responses of genes that have a zinc transcriptional regulatory element (ZTRE). It suggests that MTF-1 is high up in a hierarchy of sensors, and that its control of the expression of ZnT1 and MTs buffers the transcriptomic response to zinc [55]. A postulated mechanism of how MTF-1 functions is that a 10–50-fold difference in affinity of its zinc fingers is used for zinc sensing [56]. This direct effect of zinc on gene expression, via the zinc sensor MTF-1, is in addition to its indirect effects as a structural cofactor in hundreds of transcription factors with zinc fingers and related motifs.

The biochemistry of both MTF-1 and MTs shows that the control of zinc homeostasis interacts with numerous other cellular systems. A host of factors induce MT expression, not only with the purpose of sequestering any surplus of zinc, but also for providing enough metabolically available zinc for cellular processes. The rapid zinc binding, the mechanisms of releasing zinc, and the affinities of MTs for zinc match the requirements for controlling cellular zinc. Only now, 60 years after its discovery, with an understanding of both the quantitative aspects of zinc metabolism and the complexity of cellular homeostatic control of zinc, the role of the particular zinc/thiolate cluster chemistry of MTs in zinc metabolism, believed to be elusive, is becoming evident [15]. 

## 6. Definition of Signalling/Regulation with Regard to Zinc

It was the intent of this short account to introduce the paradigms of zinc signals and zinc signalling for some of the regulatory roles of zinc. The terms “signals” and “signalling” have been applied to describe different scenarios where zinc (II) ion transients are detectable. With some ambiguity in the use of the terms, the credibility of the field will suffer without a clear definition of the role of zinc in cellular signalling.

First, the terms zinc signals and zinc signalling should be used only where zinc ions participate as messengers in cellular signalling. The criteria for a second messenger, namely that changes of a short-lived metabolite—and a mechanism to terminate the response—lead to a rapid alteration of the activity of (an) enzyme(s), are fulfilled in the above pathways. Of course, with metal ions, the metabolite itself is not short-lived—the availability of the metal ion controls the signal. As pointed out in calcium biology, where IP_3_ is the second messenger that releases calcium from the ER, calcium is actually the third messenger. This distinction is rarely made, and calcium is generally referred to as a classical second messenger. In the same line of thought: zinc will be the fourth messenger in the signalling cascade when the calcium signal is upstream of the zinc signal. The other signalling functions discussed refer to extracellular zinc ions. Here, Zn^2+^ is a first messenger, a stimulus like a hormone binding to a receptor, when it binds to GPR39. For such an action, a zinc signal must be generated in the extracellular environment which has a buffered steady-state concentration of zinc.

Second, the terms should be reserved for transient physiological signals and not be used for non-physiological concentrations of zinc that are the result of an imbalance or a breakdown of homeostatic control. The two situations are part of a spectrum of actions, and can be distinguished only when it is known that the homeostatic system is unable to cope with the zinc concentrations. 

Third, like calcium signals, zinc signals have spatiotemporal characteristics with relatively short transients. I suggest that the terms “late” zinc signals and signalling should not be used because they are a consequence of the short-lived zinc transients, and refer to slow transcriptional, or other responses to zinc. For example, cells adjust to different physiological states requiring a re-programming that may involve gene expression. Resting, proliferating, differentiating, and apoptotic cells all have a different basal “free” zinc ion concentration [7]. 

## 7. Conclusions

The most important message is that zinc is a major cellular regulatory ion in the series of biologically redox-inert metal ions Na^+^, K^+^, Mg^2+^, Ca^2+^, Zn^2+^, making zinc regulation not a specific topic with isolated modes of actions, but a general one in cellular biochemistry/biology. The regulatory functions are in addition to its roles as a catalytic and structural cofactor in the already impressive number of zinc metalloproteins. Cellular zinc is controlled by a large network of specific proteins that interact with virtually all pathways controlling metabolism and cell fate. A most recent example is the identification of ZIP9 as an androgen receptor that is coupled to G-proteins, and mediates non-classical responses to androgens [57].

Recent developments show the field of zinc biology to be as important as the fields of iron or calcium biology. Zinc shares relatively high abundance and cellular concentrations with both. Zinc is not a trace element, but rather a mineral and major constituent of the cell. While iron biochemistry has been a forerunner in metallobiochemistry, zinc biochemistry turns out to be an even more pervasive topic. Zinc has been classified as a type 2 nutrient, such as magnesium, with a general function in metabolism, as opposed to type 1 nutrients, such as iron with more specific functions [58]. With iron, it shares the many functions in metalloproteins, and with calcium, it shares the signalling capacity. Like calcium, genetic and environmental factors that affect homeostatic control can cause zinc disorders and dysregulation, which are the cause of many diseases [59].

The physicochemical properties that make zinc ideally suited for the wide range of biological functions have been pointed out repeatedly, and the potential of the field was predicted in a now classic review published 25 years ago [60]. Likewise, the anticipation that zinc will be the calcium of the 21st century, in terms of its signalling capacities, seems to be gaining traction [61]. The time has come for the implications of zinc serving major regulatory functions in the cell to be recognized outside the relatively small community of zinc biologists.

## Abbreviations

PTP	protein tyrosine phosphatase
ZnT	zinc transporters
ZIP	Zrt, Irt-like proteins (zinc transporters)
MT	metallothionein
MTF-1	(MRE)-binding transcription factor 1
ER	endoplasmic reticulum
IP_3_	inositol triphosphate
ZnR	Zinc-sensing receptor
GPR	G-protein-coupled receptor
